# Notch1 signaling induces epithelial-mesenchymal transition in lens epithelium cells during hypoxia

**DOI:** 10.1186/s12886-017-0532-1

**Published:** 2017-08-01

**Authors:** Lei Liu, Wei Xiao

**Affiliations:** 1 Department of Ophthalmology, Shengjing Hospital, China Medical University, NO.36 Sanhao Street, Shenyang City, Liaoning Province 110004 China; 2 Department of Ophthalmology, Shenyang The Fourth Hospital of People, NO.20 Huanghe South Street, Shenyang City, Liaoning Province 110031 China

**Keywords:** Posterior capsular opacification (PCO), Epithelial-mesenchymal transition (EMT), Cobalt chloride (CoCl_2_), Hypoxia-inducible factor-1 alpha (HIF-1α), Notch1

## Abstract

**Background:**

Posterior Capsular Opacification (PCO) is one of the most common complications of cataract surgery which can result in severe visual damage. Epithelial-Mesenchymal Transition (EMT) of lens epithelium cells (LEC) is the pathological basis of PCO. Recent research showed that hypoxia acted as an inducer of EMT through a Notch1/Snail1/E-cadherin pathway. However, it remains unclear whether the Notch1/Snail1/E-cadherin pathway is involved in PCO under hypoxia.

**Methods:**

The morphology of SRA01/04 cells treating with Cobalt Chloride (CoCl_2_) was observed and the markers of EMT and Notch1/Snail1/E-cadherin pathway were analyzed by Western blot and Immunocytochemistry assay. Transwell invasion assay and Wound healing assay were used to detected the effect of p3 × FLAG-CMV-7-NICD1 transfection on the SRA01/04 cells.

**Results:**

The SRA01/04 cells lost cell polarity and cell junction culturing with CoCl_2_. The expression of Keratin, Hypoxia-inducible factor-1 alpha (HIF-1α), Notch1, Snail1were upregulated, on the other side, Fibronectin and E-cadherin were downregulated in hypoxia. Furthermore, the overexpression of Notch1 induced the expression of E-cadherin and increased the invasion and migration ability of SRA01/04 cells.

**Conclusions:**

These results suggest that Notch1/Snail1/E-cadherin pathway facilitates the EMT through HIF-1α in SRA01/04 cells during hypoxia and promotes LEC motility.

## Background

Cataract surgery is the most common ophthalmic surgical procedure in now days. However, Posterior Capsular Opacification (PCO), which can result in severe visual damage, is one of the most common complications of modern cataract surgery [1]. Close examination of lens cell populations in human donor capsular bags with implanted intraocular lenses [2–4] revealed that the Epithelial-Mesenchymal Transition (EMT) of LEC migration from the anterior capsular membrane is the pathological basis of PCO.

The lens metabolizes actively and requires nourishment to maintain its growth and transparency. However, the lens need lower energy demands, lack of aerobic respiration and consume little oxygen comparing to other tissues in the eye [5]. The anterior portion of lens absorbs the nutrition including oxygen from the anterior chamber, aqueous humor, while the oxygen supply of the posterior portion of the lens is maintained by the vitreous [6]. The oxygen tension in aqueous humor is much higher than that in the vitreous [7–9], which creates an oxygen-rich environment in the anterior portion of the lens but an oxygen-poor environment in the posterior part. The lens epithelium is physiologically located only in the oxygen-rich anterior portion of the lens. After cataract surgery, the LECs move to the posterior portion of the lens to a hypoxic microenvironment, where it may undergo EMT.

EMT is defined as a transition from epithelial cells to mesenchymal cells [10] and is an essential process not only in development [11], but also in fibrosis [12], tumor metastasis [13, 14], and wound healing [15]. In this process, epithelial cells down-regulate the expression of cell adhesion molecule (such as E-cadherin), deposit ECM proteins, dissolve cell–cell junctions, lose their apical–basal polarity, and obtain migratory and invasive behavior [16, 17]. Due to the change in oxygen environment during EMT, hypoxia has gained significant attention in the recent research of EMT [18, 19]. Variation of the microenvironmental oxygen levels and activation of hypoxic signaling pathway through Hypoxia-Inducible Factor (HIF) are regarded as significant triggers and modulators of EMT [20].

Notch1/Snail1/E-cadherin pathway is recently reported to induce EMT in several diseases [21–24]. The Notch gene was discovered by Morgan T.H.in *Drosophila melanogaster* nearly a century ago [25], the absence of which causes a ‘notched’ defective wing in drosophila. Notch signaling influenced several cellular processes, including proliferation, differentiation and apoptosis [26]. Notch1 signaling pathway also played an essential role in the growth and differentiation in developing lens [27, 28]. HIF-1α enhances Notch signaling [29] and interacts with Notch Intracellular domain, NICD [30], the intracellular activating portion of Notch. Although the activation of Notch signaling induces EMT, it remains unclear whether the Notch1/Snail1/E-cadherin pathway is involved in PCO under hypoxia. Therefore, this study investigated the involvement of Notch1/Snail1/E-cadherin signaling in EMT in LEC during hypoxia.

## Methods

### Cell culture

SRA01/04 cells (The human Lens Epithelium Cell line) was cultured in RPMI-1640 with 10% fetal bovine serum, 10 U/l penicillin G, and 100 mg/l streptomycin. Hypoxic mimic condition was generated by the treatment with Cobalt Chloride (CoCl_2_, Sigma). The RPMI-1640 medium containing 150 μM CoCl_2_ was sterilized by 0.22 μM filters and preserved at 4 °C. The control groups were cultured in the absence of CoCl_2_.

### Immunocytochemistry

SRA01/04 cells were treated with 150 μM CoCl_2_ or not_._ Cells were fixed in 4% paraformaldehyde, then permeabilized in 0.5% Triton X-100 three times. After blocking with 0.5% bovine serum albumin (BSA) for 2 h, the cells were incubated with each primary antibodies in a wet box at 4 °C overnight. The primary antibodies used were mouse anti-Keratin (1:500) and rabbit anti-Fibronectin (1:500) (Thermo Fisher, USA). On the following day, cells were incubated with secondary antibody diluted 1:500 (Alexa Fluor 488-conjugated goat anti-mouse and Alexa Fluor 594-conjugated goat anti-rabbit secondary antibody, Thermo Fisher, USA) for 2 h at room temperature. Then 5 μg/ml 4′,6′-diamidino-2-phenylindole (DAPI, Thermo Fisher, USA) was used to stain nuclei. Immunostaining was visualized by using a Nikon Eclipse 80i microscope (20 × magnification) and digitally imaged with NIS-Elements F 2.3 software package. Assessment of the staining intensity was based on the ratio of Keratin and Fibronectin to DAPI respectively. The Data presented are representative of five randomly selcected fields and five cells were measured in each field. The immunocytochemistry assay was repeated three times.

### Plasmid and transfection

The p3 × FLAG-CMV-7-NICD1 plasmid was obtained from Raphael Kopan (Addgene plasmid # 20183). The SRA01/04 cells were transfected with 3 μg specific p3 × FLAG-CMV-7-NICD1 for 24 h, using Lipofectamine™ LTX reagent(Invitrogen) in serum-free RPMI-1640 medium according to the manufacture’s instruction. Control SRA01/04 cells were transfected with p3 × FLAG-CMV-7(Sigma). Western blot was used to verify the efficiency of transfection.

### Western blot and antibodies

Different groups of SRA01/04 cells were harvested and lysed in using cell lysis buffer (Beyotime, China). The lysed proteins were separated by SDS-PAGE and transferred onto a polyvinylidene fluoride membrane. After blocking, the membrane was incubated with primary antibodies (Notch1 1:1000, Snail 1:1000, E-cadherin, Keratin, Fibronectin and GAPDH 1:500), followed by correspongding horseradish peroxidase-conjugated secondary antibodies (1:2000) for 90 min at RT. The protein bands were detected by enhanced chemiluminescence (SuperSignal West Pico. Thermo, USA) and recorded using a DNR Bio-Imaging System (Israel). The ratio of the optical densities between Notch-1, Snail, E-cadherin, Keratin, Fibronectin and GAPDH represent the relative protein expression. The primary antibodies against Notch1 and Snail were from Cell Signaling Technology (USA). The primary antibodies against E-cadherin, Keratin, Fibronectin, GAPDH and the secondary antibody were form Bioss (China).WB was repeated three times and three samples were used in each time.

### Transwell invasion assay

24-well Transwell chambers (8-μm pore size, Corning) were used in transwell invasion assay. The transwell filters were covered with matrigel (50 μl) on the upper surface of the polycarbonic membrane. At 24 h after transfection, 4 × 10^4^ cells were seeded in the upper chamber in RPMI1640 medium without serum. The lower chamber was filled with the same medium with 10% FBS. 48 h later, non-invading cells were cleaned. The invading cells were fixed in 4% paraformaldehyde and stained by Crystal Violet. The number of invaded cells was counted in three randomly selected fields at high magnification. Data presented are representative of three individual wells and transwell invasion assay was repeated three times.

### Wound healing assay

SRA01/04 cells were cultured and transfected in a 6-well plate. A 10 μl pipette tip was used to make a straight scratch through the monolayers. In the subsequent period, cells migrated into the wound surface. The scratch width was used to represent the migrating ability of SRA01/04 cells. 24 h later, the scratch width was measured in five randomly selected fields. Data presented are repeated three times.

### Statistical analysis

All the statistical analyses were performed with SPSS13.0 using one-way ANOVA followed by S-N-K post-hot test. *p* < 0.05 is considered significantly difference.

## Results

### Effect of CoCl_2_ treatment on the SRA01/04 cells

**Fig. 1:**
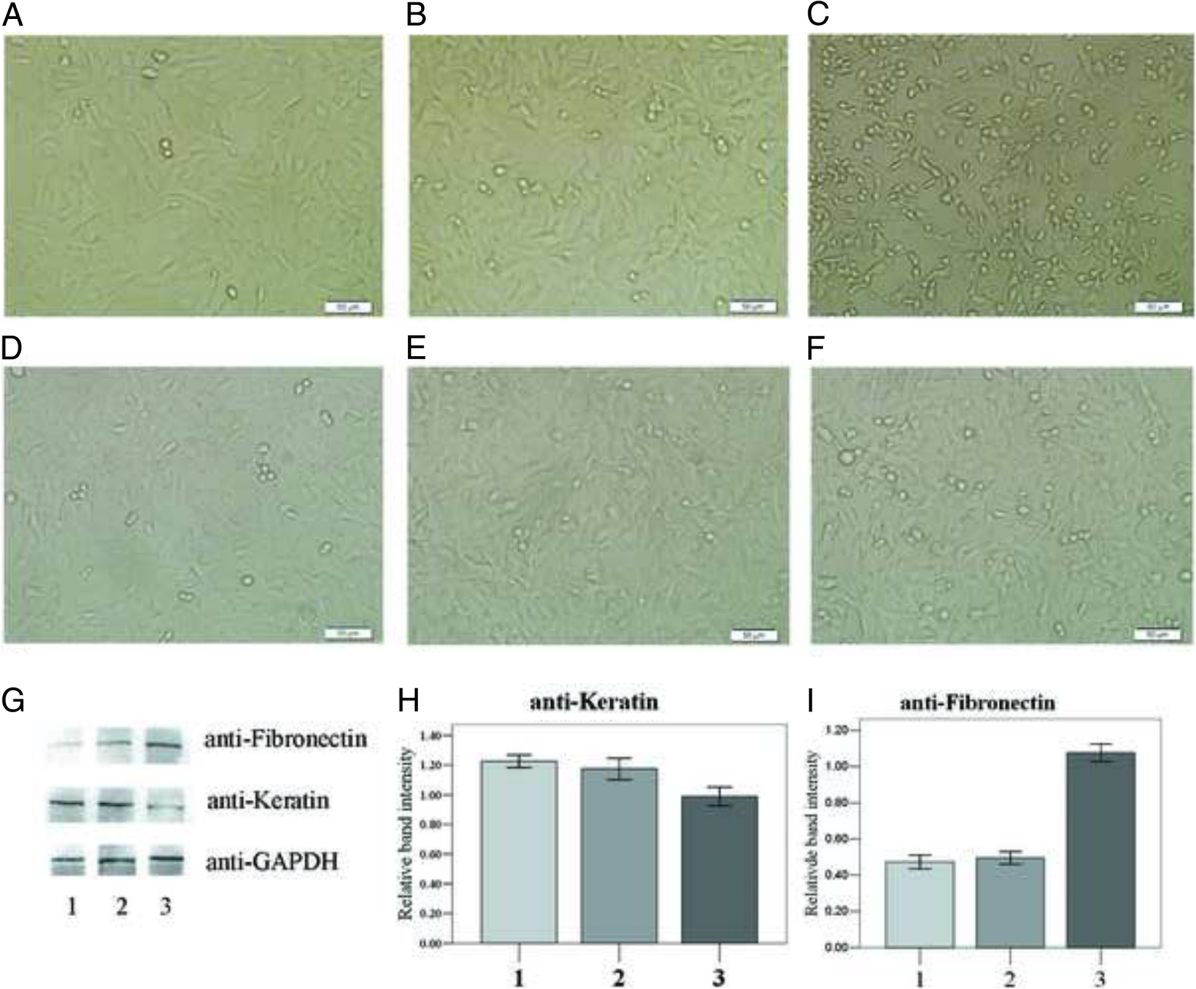
Effect of CoCl_2_ treatment on the SRA01/04 cells. **a**-**f** Transmission images of SRA01/04 cultured in the presence (**a**, **b**, **c**) and absence (**d**, **e**, **f**) of 150 μM CoCL_2_for 12 h (**a**, **d**), 24 h (**b**, **e**) and 48 h (**c**, **f**). The morphology of the SRA01/04cells at 48 h after CoCL_2_treatment (**c**) appeared no cell polarity and lost cell junction. **g**-**i** western blot images (**g**) and average relative band intensity of Keratin (**h**) and Fibronectin (**i**) in SRA01/04 cells cultured with 150 μM CoCL_2_ for 12 h (group1), 24 h (group2), 48 h (group3). At 48 h of CoCL_2_ stimulation, the expression of Keratin decreased most significantly (*P* < 0.05), and Fibronectin increased most significantly (*P* < 0.05)

**Fig. 2:**
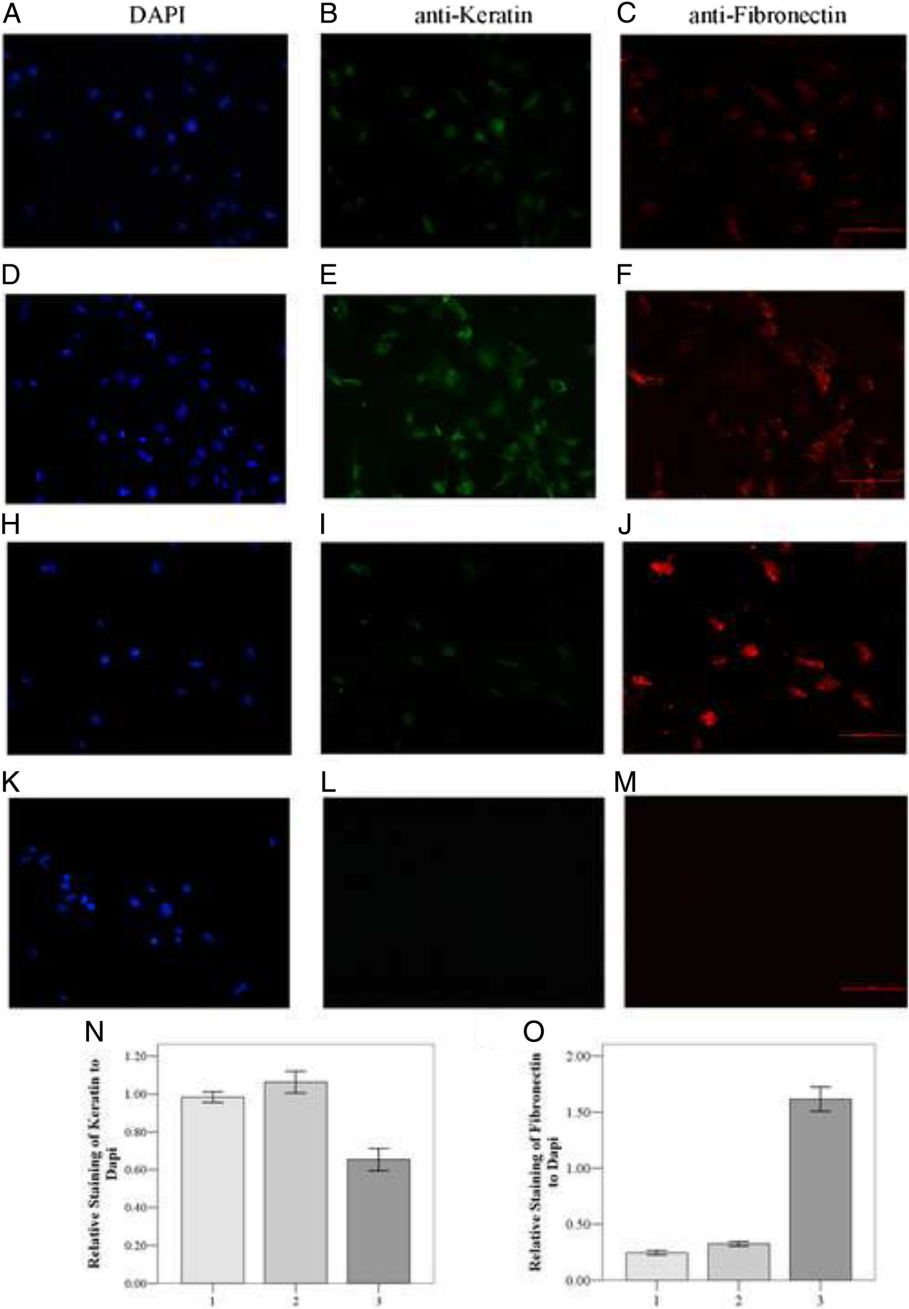
Immunofluorescence microscopy analysis of Keratin degradation and Fibronectin enhancement by CoCl2 stimulated on SRA01/04 cells. Human SRA01/04 cell monolayers were cultured on coverslips and 48 h after CoCl2 treatment, coverslips were analyzed by immunofluorescence microscopy. *Red* represents Fibronectin and *green* represents Keratin. Images of SRA01/04 cells: 12 h cultured without CoCl_2_ treatment (**a**-**c**), 48 h without CoCl_2_ treatment (**d**-**f**); 48 h treated with CoCl_2_ (**h**-**j**). treated only with second antibodies (**k**-**m**). **n** Quantification of the percentage of Keratin-positive SRA01/04 cells, relative to DAPI-stained nuclei. **o** Quantification of the percentage of Fibronectin-positive SRA01/04 cells, relative to DAPI-stained nuclei

CoCl_2_ is mostly used to mimic hypoxic conditions in cell cultures. we first examined the morphology changes of SRA01/04 cells cultured in CoCl_2_ for 12,24 and 48 h (Fig. 1a–c). The most obvious changes happened when cells were cultured in CoCl_2_ for 48 h.They had no cell polarity and lost cell junction (Fig. 1c), the characteristics of mesenchymal cells. In addition, 48 h CoCl_2_ treatment induced a significant decrease in the expression of Keratin, and an increase in the expression of Fibronectin (*p* < 0.05) (Fig. 1g–i). So the expression of Keratin and Fibronectin in SRA01/04 cells were also tested in immunocytochemical assay especially on 48 h (Fig. 2). As a result, the red fluorescence (Fibronectin) was most obvious (Fig. 2j, *p*<0.05) and the green fluorescence (Keratin) weakened distinctly (Fig. 2i, *p*<0.05) in SRA01/04 cells treated by CoCl_2_ for 48 h.

### Expression of HIF-1α,Notch1, Snail1 and E-cadherin in SRA01/04 cells following treatment with cobalt chloride

**Fig. 3:**
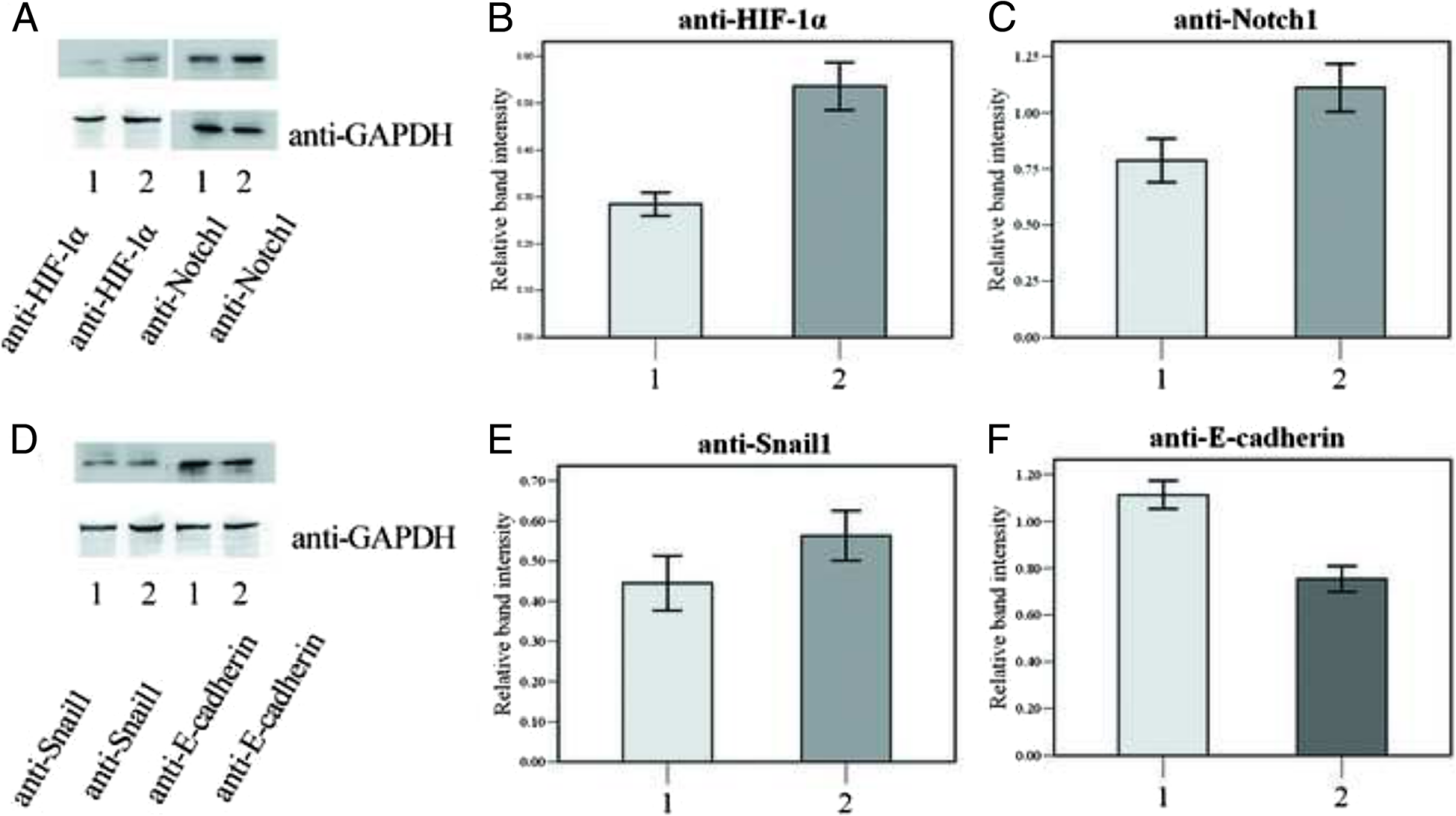
Protein expression of HIF-1α, Notch1, Snail and E-cadherin under the treatment with Cobalt chloride. Western blot images (**a** & **d**) and average relative band intensity of HIF-1α (**b**), Notch1 (**c**), Snail1(**e**) and E-cadherin (**f**) in SRA01/04 cells cultured in the presence (group2) or absence (group1) of 150μMCoCL_2_for 48 h

Western Blot analysis showed that the expression of HIF-1α (Fig. 3a & b), Notch1 (Fig. 3a & c), Snail1 (Fig. 3d & e) all increased after 48 h CoCl_2_treatment in SRA01/04 cells, (*p* < 0.05) while the E-cadherin expression was decreased (Fig. 3d & f, *p*<0.05)

### Overexpression of Notch1 decreased expression of E-cadherin via activation of Snail1

**Fig. 4:**
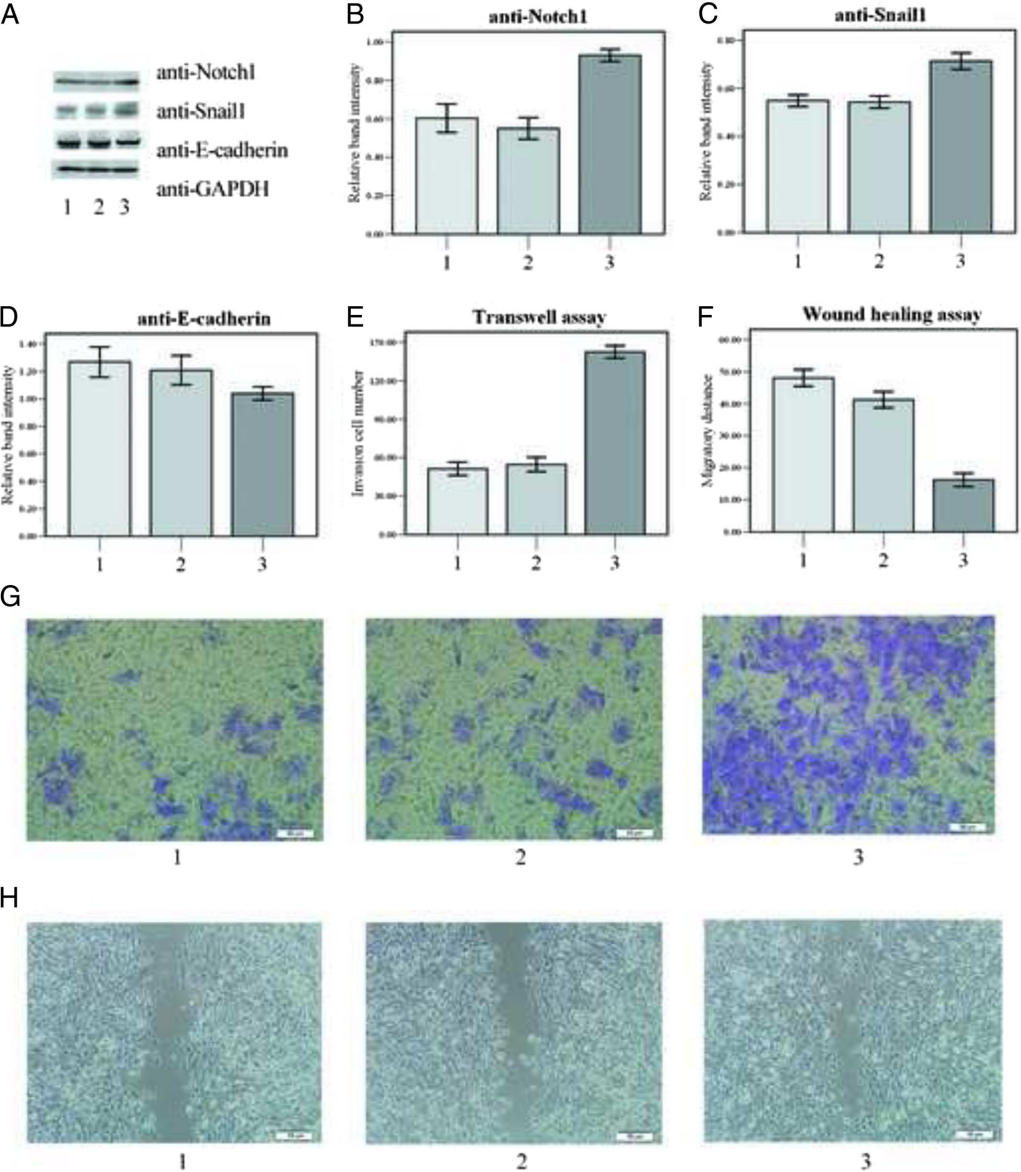
Notch1/Snail1/E-cadherin pathway increased the invasion and migration ability of SRA01/04 cell. **a**-**d** WB image (**a**) and average relative band intensity of Notch1(**b**), Snail1 (**c**) and E-cadherin (**d**) showed the overexpression of Notch1 24 h after transfected with p3 × FLAG-CMV-7-NICD1 (group 3) and increased expression of Snail1 and decreased expression of E-cadherin compared with negative control either transfected with empty vector p3 × FLAG-CMV-7 (group 1) or non-transfected cells (group 2). **e** & **g** Representative images (**g**) and average number of invaded cells in the lower chamber during transwell assay 24 h after transfection of empty vector (group1), non-transfected (group2) and transfected with Notch1-NICD (group 3). **f** & **h** Representative images (**h**) and average scratch width in wound healing assay following the same transfection group as **e** & **g**

Notch1 was then overexpressed in SRA01/04 cells to examine the effect of Notch1/Snail1/E-cadherin activation. SRA01/04 cells were transfected with p3 × FLAG-CMV-7-NICD1 (Fig. 4 group3), using cells transfected with empty vector p3 × FLAG-CMV-7 (Fig. 4 group1) and non-transfected cells (Fig. 4 group2) as controls. The protein level of Notch1 was confirmed to be upregulated in group 3 (Fig. 4a & b), compared with group1 and group2. Snail1 was correspondingly upregulated (Fig. 4a & c) and E-cadherin was downregulated (Fig. 4a & d) in group3.

### Overexpression of Notch1 enhanced the migration of SRA01/04 cells

Transwell assay was performed to study the effect of Notch1 on migration ability of SRA01/04 cells. As shown in Fig. 3g, the number of cells invaded across the polycarbonate membrane significantly increased in group3 (142.3 ± 4.9) compared with group1 (54.6 ± 5.5) and group2 (51.2 ± 5.2) (Fig. 4e *P* < 0.05). But the difference between group 1 and group 2 was not significant (*P* > 0.05).

Wound healing assay was also used to examine the effect of Notch1 on cell migration. The scratch width, 24 h post injury reflects the cell migration ability. As shown in Fig. 4h, the narrowest width was founded in group3 (16.2 ± 2.1 μm) compared with group1 (48.0 ± 2.6 μm) and group2 (41.2 ± 2.5 μm) (*P* < 0.05). No significant difference was found between group 1 and group 2 (Fig. 3f, *P*>0.05).

## Discussion

Harold Ridley, a British ophthalmologist, made the first intraocular lens implantation to treat cataract in 1949. From then on, clinical treatment of cataract has improved continuously and studies on the pathogenesis of cataract have advanced significantly. However, PCO is still a common complication of cataract surgery with unknown cause, which is affecting the overall results of cataract treatment. The incidence and severity of PCO correlates to the use of surgical techniques, intraocular lens optic edge designs and intraocular lens materials in clinic [31]. On a biochemical level, the proliferation, migration and abnormal differentiation of residual lens epithelial cells and fibers in the capsular bag have been involved in the pathogenesis of PCO. Among these factors, EMT was recognized to play an critical role in the development of PCO.

Hypoxia has been associated with EMT through potentiating Notch signaling [32–34]. HIF-1a also played an essential role in the EMT under hypoxia. Since HIF-1a interacts with Notch-NICD, this suggest that HIF-1a may induce EMT through activating the Notch signaling pathway during hypoxia.

In this study, we found that EMT occurs in SRA01/04 cells induced by CoCl_2_.CoCl_2_ indicating that hypoxia led to profound morphological changes in LEC. The upregulatin of Notch1,Snail1 and HIF-1a in SRA01/04 cells following treatment suggest that both the Notch1/Snail1/E-cadherin signaling and HIF-1a are involved in EMT induced by hypoxia.

Former studies have indicated that Snail expression can be directly induced by the Notch signaling pathway [35, 36]. However, this process was never examined in LEC. In addition, the biological function of Notch1/Snail1/E-cadherin in LEC has never been reported either. In this present study, we confirmed that the expression of Snail is upregulated upon over expression of Notch1 in SRA01/04 cells, suggesting a tight correlation between the regulations of the two proteins in LEC. The enhanced migration ability in SRA01/04 cells following Notch1 overexpression is consistent with the transition of LEC from epithelial cells to mesenchymal cells (typically high mobility) and provides strong evidence on the involvement of Notch1 in EMT in LEC.

Therefore, we hypothesize that the residue LEC migrated to the posterior capsule after cataract surgery where the lower oxygen level initiates the Notch1 pathway through HIF-1a in LEC, and promotes the transformation of LEC into mesenchymal cells. This notion is also supported by another research from reverse [37]. In this research, active oxygen processing intraocular lenses were used to prevent PCO.

## Conclusions

In summary, we showed initially that Notch1/Snail1/E-Cadherin pathway may facilitate the EMT and promote cell motility, possibly through HIF-1a in SRA01/04 cells under hypoxia, which may participate in PCO pathogenesis. Further studies are needed to determine a causative effect of HIF-1a and Notch1 in the occurrence of EMT during hypoxia using gene knockdown or knockout both in cell lines and in animal models. The elucidation of the molecular mechanism of EMT through Notch1/Snail1/E-Cadherin pathway may provide new molecular targets for the prevention and treatment of patients with PCO.

## Abbreviations

BSA: bovine serum albumin
CoCl_2_: Cobalt Chloride
DAPI: 4′,6′-diamidino-2-phenylindole
EMT: Epithelial-Mesenchymal Transition
HIF: Hypoxia-Inducible Factor
LEC: lens epithelium cells
NICD: Notch Intracellular domain
PCO: Posterior Capsular Opacification
